# Estrous Cycle-Dependent Phasic Changes in the Stoichiometry of Hippocampal Synaptic AMPA Receptors in Rats

**DOI:** 10.1371/journal.pone.0131359

**Published:** 2015-06-29

**Authors:** Hirobumi Tada, Mayu Koide, Wakana Ara, Yusuke Shibata, Toshiya Funabashi, Kumiko Suyama, Takahisa Goto, Takuya Takahashi

**Affiliations:** 1 Department of Physiology, Yokohama City University Graduate School of Medicine, 3–9 Fukuura, Kanazawa-ku, Yokohama, 236–0004, Japan; 2 Department of Physiology, St. Marianna University School of Medicine, 2-16-1 Sugao, Miyamae-ku, Kawasaki, 216–8511, Japan; 3 Department of Anesthesiology, Yokohama City University Graduate School of Medicine, 3–9 Fukuura, Kanazawa-ku, Yokohama, 236–0004, Japan; Institute for Interdisciplinary Neuroscience, FRANCE

## Abstract

Cognitive function can be affected by the estrous cycle. However, the effect of the estrous cycle on synaptic functions is poorly understood. Here we show that in female rats, inhibitory-avoidance (IA) task (hippocampus-dependent contextual fear-learning task) drives GluA2-lacking Ca^2+^-permeable AMPA receptors (CP-AMPARs) into the hippocampal CA3-CA1 synapses during all periods of the estrous cycle except the proestrous period, when estrogen levels are high. In addition, IA task failed to drive CP-AMPARs into the CA3-CA1 synapses of ovariectomized rats only when estrogen was present. Thus, changes in the stoichiometry of AMPA receptors during learning depend on estrogen levels. Furthermore, the induction of long-term potentiation (LTP) after IA task was prevented during the proestrous period, while intact LTP is still expressed after IA task during other period of the estrous cycle. Consistent with this finding, rats conditioned by IA training failed to acquire hippocampus-dependent Y-maze task during the proestrous period. On the other hand, during other estrous period, rats were able to learn Y-maze task after IA conditioning. These results suggest that high estrogen levels prevent the IA learning-induced delivery of CP-AMPARs into hippocampal CA3-CA1 synapses and limit synaptic plasticity after IA task, thus preventing the acquisition of additional learning.

## Introduction

The estrous cycle can affect mental state and activity [1]. For example, women exhibit mild impairments in spatial tasks during the follicular phase of the menstrual cycle, when estrogen levels are elevated [2]. However, how the estrous cycle affects synaptic function is not well understood. Excitatory synaptic transmission in the nervous system is mediated by the actions of glutamate through ion-permeable AMPA receptors (AMPARs) [3]. Synaptic trafficking of AMPARs appears to be a major mechanism underlying long-term potentiation (LTP) in vitro [4] and experience-driven synaptic potentiation in vivo [5–15]. In the developing barrel cortex, AMPARs are delivered into layer 4-layer 2/3 synapses, where they contribute to establish a functional whisker-barrel map [7,8,11,13]. Hippocampus-dependent contextual fear conditioning drives AMPARs into the hippocampal CA3-CA1 synapses, and this process is required for the formation of fear memories [10,15,16]. Furthermore, in vivo experience as well as LTP induces the switch from GluA2-containing to GluA2-lacking AMPARs, known as calcium-permeable AMPARs (CP-AMPARs) and to be crucial for establishing synaptic plasticity [13,17].

Here we found that in female rats, inhibitory-avoidance (IA) tasks, which are hippocampus-dependent contextual fear-learning tasks, delivered CP-AMPARs into the hippocampal CA3-CA1 synapses during all periods of the estrous cycle except the proestrous (ProE) period, when the estrogen level is high. The induction of LTP was prevented after IA task at the CA3-CA1 synapses of female rats in the ProE period, while intact LTP was expressed after IA learning in other estrous period.

In ovariectomized (OVX) rats that were not treated with estrogen, CP-AMPARs were increased at the synapses after IA task. When OVX rats were treated with estrogen, IA task failed to drive CP-AMPARs into the synapses. In the absence of estrogen treatment, LTP was induced at CA3-CA1 synapses in OVX animals after IA task; however, LTP was not induced after IA task in the presence of estrogen treatment. Consistent with this finding, intact female rats did not acquire hippocampus-dependent Y-maze task after IA conditioning during the ProE period, but were able to learn Y-maze task after IA task during other estrous period. These results suggest that high estrogen levels prevent the IA-learning-induced delivery of CP-AMPARs into hippocampal CA3-CA1 synapses and restrict synaptic plasticity after IA task, leading to the prevention of further acquisition of learning tasks.

## Materials and Methods

### Ethics Statement

All experiments were conducted according to the Guide for the Care and Use of Laboratory Animals (Japan Neuroscience Society) and the Guide for the Yokohama City University. All animal experiments were approved by the Animal Care and Use Committee of Yokohama City University (authorization number: F-A-14-029). All surgical procedures were performed under anesthesia, and every effort was made to minimize suffering.

### Animals

Female 8-week-old Wistar rats were purchased from Charles River (Yokohama, Japan). The rats were maintained at 24–28°C under controlled lighting conditions (light on from 5:00 to 19:00) with food and water *ad libitum*. Daily vaginal smears were taken to determine the estrous day. OVX rats were prepared as described previously [18]. Estradiol treatment was administered under anesthesia by isoflurane gas; a silastic tube (inner diameter 1.5 mm; outer diameter 2.5 mm; length 25 mm) containing 20% 17β-estradiol (Sigma Chemical Co., USA) was implanted subcutaneously 3 days before conducting experiments.

### Electrophysiology

Rats were anesthetized with an isoflurane-oxygen mixture, and the brain was removed and quickly transferred into gassed (95% O_2_ and 5% CO_2_) ice-cold dissection buffer as described previously [15]. Coronal brain slices were cut (400 μm, Leica VT1000) in dissection buffer and incubated in artificial cerebrospinal fluid (ACSF) as described previously [15,19]. Patch-recording pipettes (3–7 MΩ) were filled with intracellular solution as described previously.

For rectification experiments, the recording chamber was perfused with ACSF containing 0.1 mM picrotoxin, 4 μM 2-chloroadenosine, and 0.1 mM D,L-APV at 22–25°C. Whole-cell recordings were obtained from CA1 pyramidal neurons in the rat hippocampus using a Multiclamp 700B (Axon Instruments). Bipolar tungsten stimulating electrodes were placed in mossy fibers, and the stimulus intensity was increased until a synaptic response with an amplitude > ~10 pA was recorded. Synaptic AMPA receptor-mediated currents at –60 mV and +40 mV were averaged over 30–50 trials, and their ratio was used as an index of rectification.

LTP was induced by pairing 3-Hz stimulation with depolarization of the postsynaptic neuron to +0 mV for 90 sec. Recordings were maintained for at least 40 min after pairing. The excitatory postsynaptic current (EPSC) amplitude throughout the recording was always normalized to the average baseline amplitude before pairing. Experiments were excluded from analysis if changes in transmission were observed in unpaired control pathways. LTP experiments were performed during 9:00–13:00.

### Western blotting

Hippocampal samples were rapidly dissected and stored at -80°C. Synaptoneurosome fractions were prepared as previously described [20]. Frozen samples were homogenized in ice-cold homogenization buffer (10 mM Hepes, 1.0 mM EDTA, 2.0 mM EGTA, 0.5 mM DTT, 0.1 mM PMSF, 10 mg/L leupeptin, and 100 nM microcystin). The tissue was homogenized in a glass/glass tissue homogenizer. The homogenates were passed through two 100-μm-pore nylon mesh filters, and then through a 5-μm-pore filter. The filtered homogenates were centrifuged at 3600×g for 10 min at 4°C. The resulting pellets were resuspended in 100 μL of boiling homogenization buffer with 1% SDS, followed by immunoblotting. The signal intensity of each band was measured by MultiGauge (Fujifilm). The net signal was obtained by subtracting the background signal obtained from the region adjacent to the band. Antibodies to GluA1 (Millipore: 04–855), GluA2 (Abcam: ab20673), and GAPDH (CST: 2118) were used.

### Golgi staining

Rats were anesthetized with an isoflurane-oxygen mixture. The brain hemispheres were removed. The impregnation procedure was carried out using FD Rapid GolgiStain kit (FD NeuroTechnologies) according to the manufacturer’s protocol, and then sectioned at 200 μm and mounted on gelatin-coated slides. The sections were then stained, dehydrated, and coverslipped using Permount (Fisher).

### Golgi-image analysis

The dendritic spine density of pyramidal neurons in the CA1 dorsal hippocampus was analyzed by counting the tertiary apical dendrites under a Keyence BZ9000 microscope. Spines were morphologically characterized under ×120 magnification (Keyence BZ9000) and classified as filopodia, stubby, or mushroom shaped spines [21]. Spines were classified as filopodia if the spine’s length was greater than its uniform diameter, as stubby if the diameter was similar to the length, and as mushroom if the diameter of the head was much greater than the diameter of the neck. Spine density was normalized as the number of spines per 10 μm of dendrite length.

### Inhibitory Avoidance Learning

On the training day, rats were moved into an electromagnetic- and sound-shielded room containing an IA-training apparatus: a two-chambered Perspex box with a lighted safe side and a dark shock side separated by a trap door. During training, rats were placed in the safe chamber facing a corner opposite the trap door. After the opening of the door, rats entered into the dark chamber at will. The latency to enter the novel dark box was measured as a behavioral parameter (i.e., latency before IA learning). The door was closed 2 sec after the animal entered the dark chamber, and a scrambled electrical foot-shock (2 sec, 0.8 mA) was applied via electrified steel rods in the floor of the box [15]. Rats were returned to their home cages after 10 sec in the dark compartment. The rats were returned to the safe chamber of the Perspex box 30 min later, and the latency to enter the experienced dark box was measured as a learning performance (i.e., latency after IA learning).

For experiments with a second IA training session, we used different-shaped box on the safe side and a 2 sec, 0.2 mA scrambled electrical foot-shock in the dark box.

### Y-maze task

The Y maze was used to assess normal hippocampus-dependent navigation behaviors. The rat was placed in a Y-shaped maze for 5 min. Number and sequence of arm entries were recorded. Alternation behavior was defined as consecutive entries into each of the three arms without repetition. The alternation percentage was calculated as the number of alternations divided by the total number of entries.

### Statistics

Means were compared by the Mann-Whitney U test and Student’s unpaired *t* test. Data are presented as means and SEM unless otherwise noted.

## Results

### IA drives CP-AMPARs into CA3-CA1 synapses during estrous cycle other than the proestrous period

**Fig 1:**
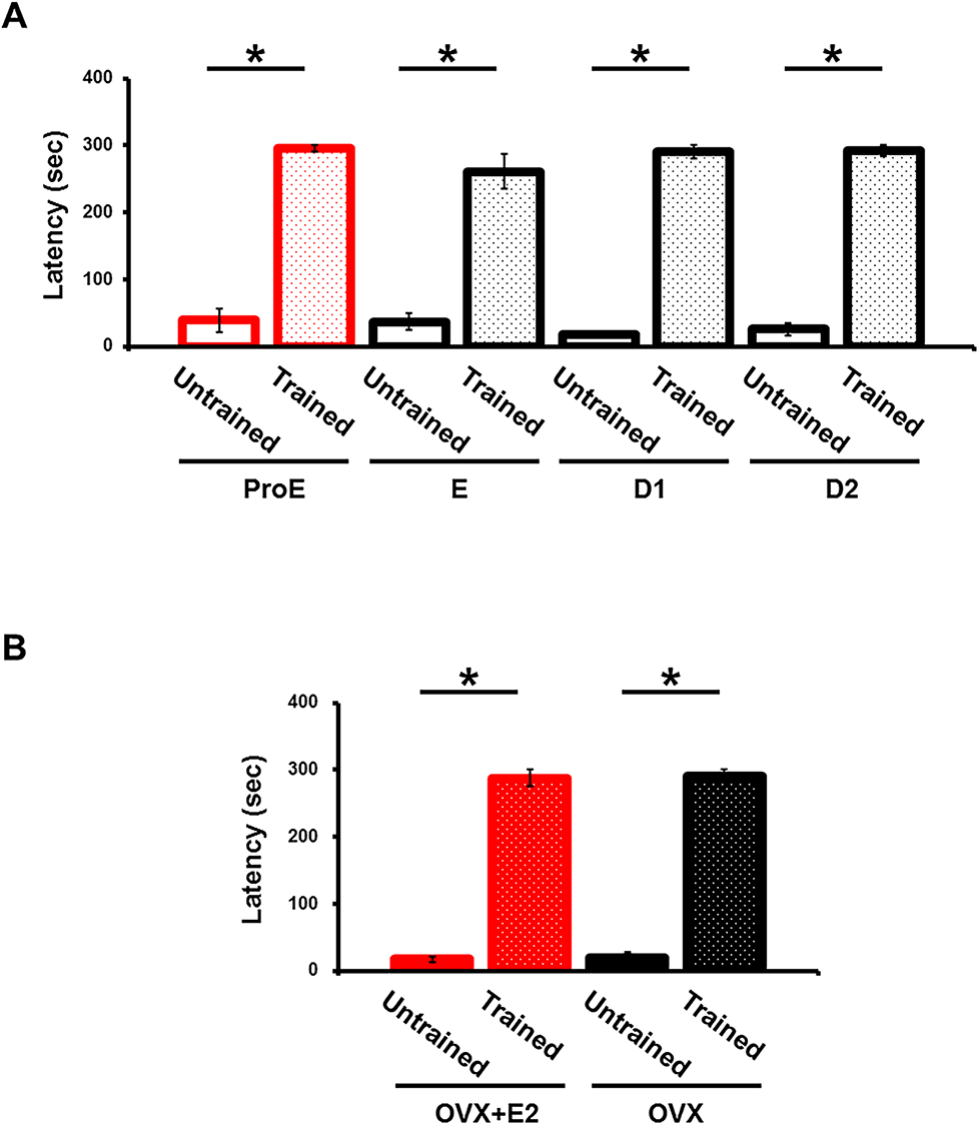
Acquisition of IA learning during the estrous cycle. Latency before entering the dark side of the box was consistently longer in IA-conditioned rats. (A) Rats successfully acquired IA memory throughout the estrous cycle. ProE (proestrous), n = 4; E (estrous), n = 6; D1 (diestrous day 1), n = 5; D2 (diestrous day 2), n = 6. (B) OVX rats successfully acquired IA memory with (n = 5) or without (n = 5) estrogen (E2) priming. *P < 0.05, unpaired Student’s *t* test. Error bars represent SEM.

To examine estrous cycle–dependent phasic changes in the AMPARs content of hippocampal synapses during task learning, we applied a hippocampus-dependent contextual fear–learning paradigm using an IA task, since IA is known to drive GluA1-containing AMPARs into the hippocampal CA3-CA1 synapses [15]. In this paradigm, rats are allowed to cross from a light box to a dark box where an electric foot shock is delivered. Thus, the rats learn to avoid the dark box and stay in the light box, which is not their natural preference. The acquisition of contextual memories can be evaluated as the tendency to avoid the dark box. Rats successfully acquired IA memory throughout the estrous cycle (Fig 1A).

**Fig 2:**
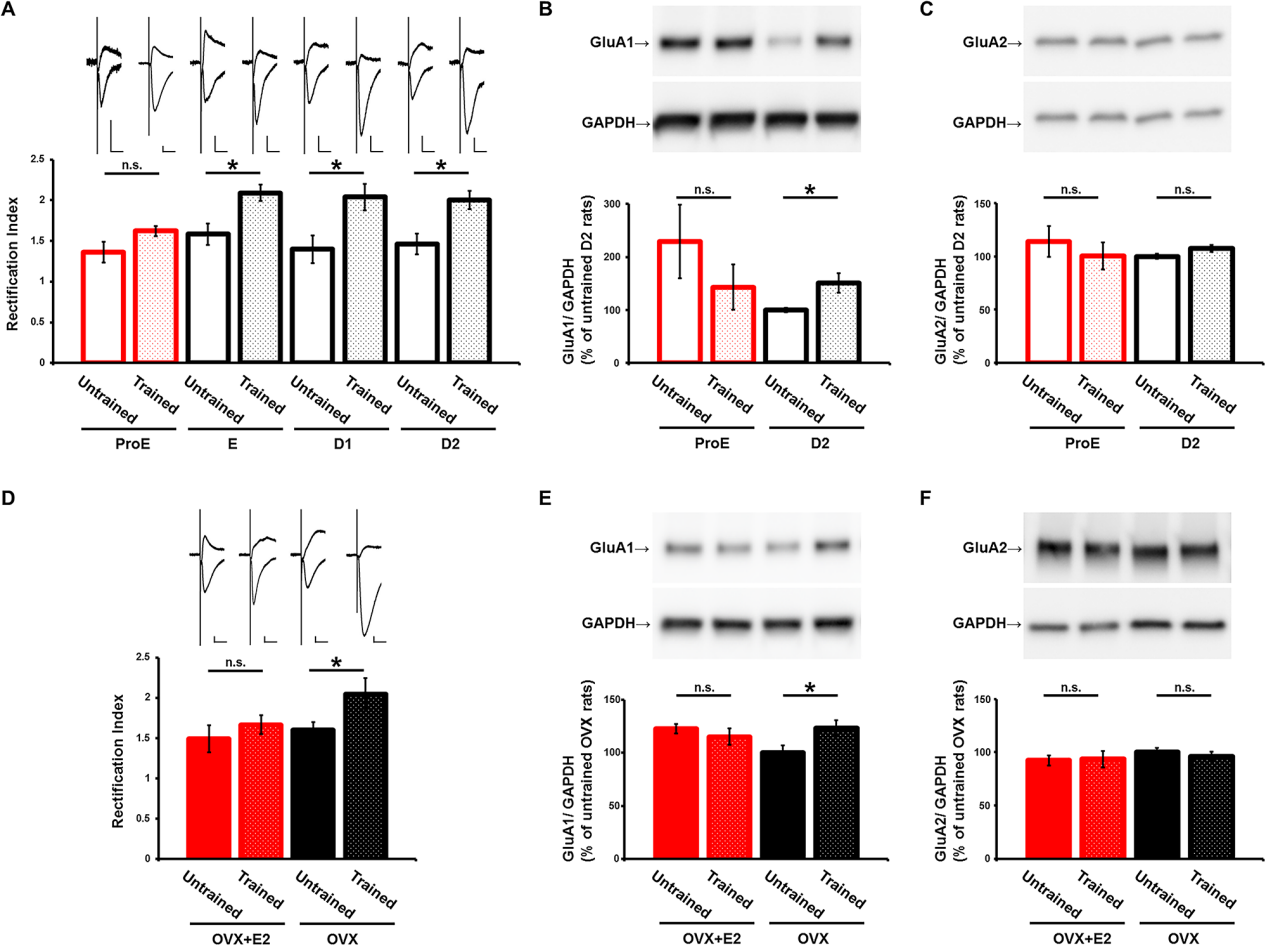
Changes in learning-driven synaptic delivery of CP-AMPARs in CA1 pyramidal neurons during the estrous cycle. (A) There was no difference in rectification in between untrained (n = 13) and IA-trained (n = 32) ProE rats. However, rectification increased notably after IA conditioning during other periods of the estrous cycle (untrained/ IA-trained: E, n = 11/ 32; D1, n = 7/ 16; D2, n = 9/ 28). (D) Rectification increased after IA conditioning in OVX rats that were not primed with E2 (untrained, n = 19; IA-trained, n = 16), but rectification did not change after IA conditioning in the presence of E2 (untrained, n = 13; IA-trained, n = 11). Scale bars: vertical, 20 pA; horizontal, 20 ms. (B, E) GluA1 and (C, F) GluA2 content in the synaptoneurosome fraction obtained from the rat dorsal hippocampus in ProE (untrained, n = 6; IA-trained, n = 6), D2 (untrained, n = 11; IA-trained, n = 16), and OVX rats with (untrained, n = 7; IA-trained, n = 7) or without E2 priming (untrained, n = 7; IA-trained, n = 7). *P < 0.05; Mann-Whitney U test (A, D) or unpaired Student’s *t* test (B-F). Error bars represent SEM.

We obtained acute brain slices 30 min after conditioning with the IA task, and recorded synaptic transmissions from CA3 to CA1 in the dorsal hippocampus. CP-AMPARs display pronounced inward rectification due to voltage-dependent blocking of the channel pore by polyamines at positive membrane potentials [17]. Although we did not observe any difference in rectification (response at -60mV/at +40mV) before or after IA learning during the ProE period, rectification increased prominently after IA learning during other periods of the estrous cycle (Fig 2A), indicating that IA learning increased the synaptic CP-AMPAR content during periods of the estrous cycle other than the ProE period. Consistent with this finding, we detected an IA-induced increase in GluA1 content in the synaptoneurosome fraction obtained from the dorsal hippocampus during periods other than the ProE period (Fig 2B). We detected no statistical difference in GluA1 content of hippocampal synaptoneurosome fractions obtained from untrained animals during the ProE versus the diestrous day 2 (D2) period (Fig 2B). In both the ProE and D2 periods, there was no difference in the GluA2 content of hippocampal synaptoneurosome fractions obtained from untrained or IA-trained animals (Fig 2C). These findings indicate that IA learning drives CP-AMPARs into the synapses at periods other than the ProE period.

### Increased estrogen prevents IA-driven synaptic CP-AMPARs delivery

To examine whether the transient increase in estrogen level at the ProE period could prevent the IA-learning-induced insertion of CP-AMPARs into the synapses, OVX rats were primed with estrogen at a dose that induced a luteinizing hormone (LH) surge. OVX rats successfully acquired IA memory with and without estrogen priming (Fig 1B). Rectification increased after IA learning in OVX rats that were not treated with estrogen, but there was no difference in rectification after IA learning in the presence of estrogen treatment (Fig 2D). IA learning increased the GluA1 content in the synaptoneurosome fraction obtained from the dorsal hippocampus of OVX rats that were not primed with estrogen, but this increase was not seen in estrogen-primed OVX rats (Fig 2E). We detected no difference of GluA2 content in the synaptoneurosome fraction of the hippocampus obtained from untrained or IA-trained OVX animals, whether unprimed or estrogen-primed (Fig 2F). These findings supported the idea that elevated estrogen blocks the IA-induced synaptic delivery of CP-AMPARs.

### LTP induction is prevented after IA task during the proestrous period

**Fig 3:**
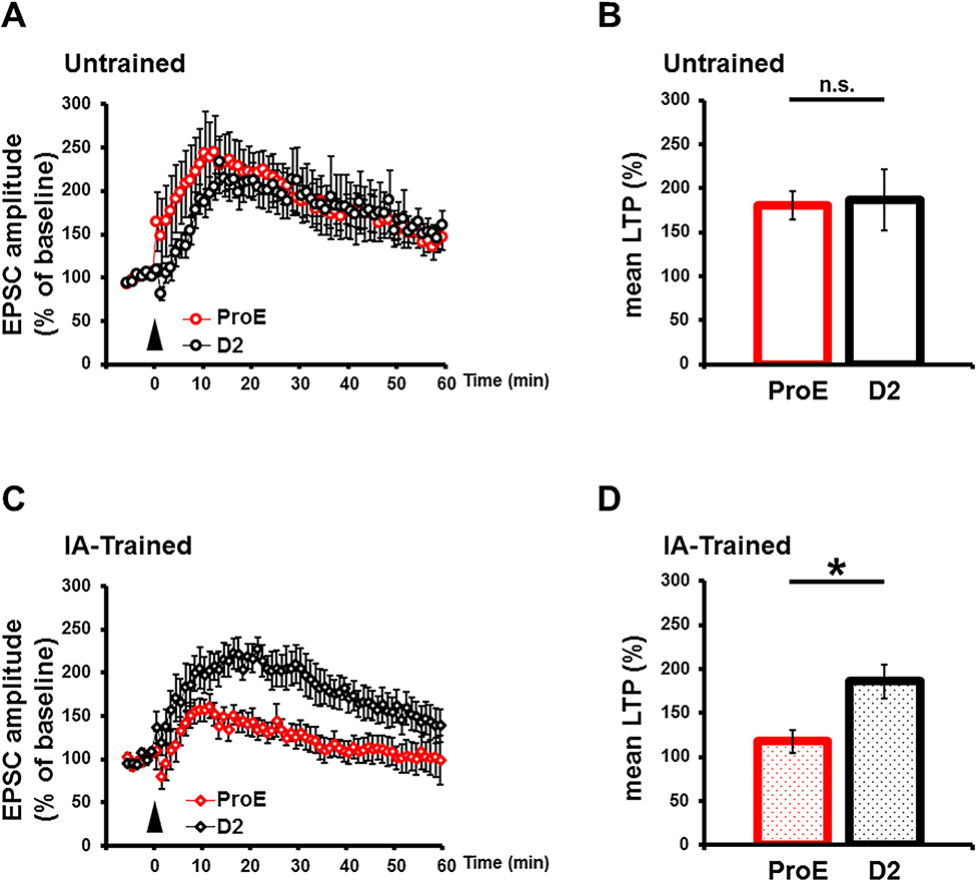
LTP was attenuated after IA task in ProE rats. (A, B) LTP was induced at hippocampal CA3-CA1 synapses from untrained ProE (n = 8) and D2 (n = 6) rats, and from (C, D) IA-trained D2 rats (n = 6), but not in synapses from IA-trained ProE rats (n = 6). The mean amplitude 30–40 min after LTP induction was normalized to the baseline amplitude. *P < 0.05, unpaired Student’s *t* test. Error bars represent SEM.

Synaptic CP-AMPARs are important for the expression of LTP [17]. To examine whether LTP is affected by difference of synaptic AMPARs content after IA learning during the estrous cycle, we induced LTP after an IA task using a paired protocol. LTP was successfully induced after IA learning during periods other than the ProE period, during which it was significantly attenuated (Fig 3C and 3D). Intact LTP was observed before the IA task even during the ProE period (Fig 3A and 3B). Thus, the capacity for synaptic plasticity after IA learning is limited during the ProE period compared to other period of the estrous cycle.

**Fig 4:**
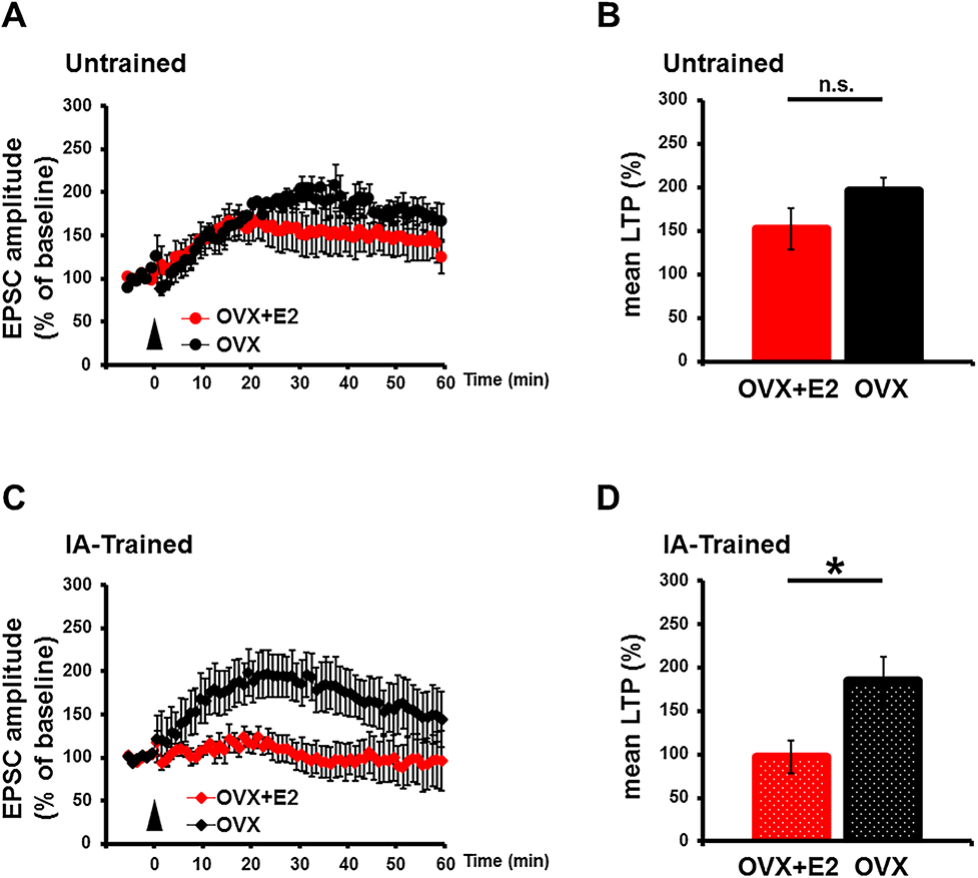
E2 priming prevented LTP after IA task in OVX rats. (A, B) LTP was induced at hippocampal CA3-CA1 synapses from untrained OVX rats with (n = 8) or without (n = 6) E2 priming. (C, D) LTP was also induced in IA-trained OVX rats without E2 (n = 8), but not in IA-trained OVX rats primed with E2 (n = 7). The mean amplitude 30–40 min after LTP induction was normalized to the baseline amplitude. *P < 0.05, unpaired Student’s *t* test. Error bars represent SEM.

To further examine the role of estrogen on LTP induction after IA conditioning, we induced LTP at the hippocampal CA3-CA1 synapses of non-primed and estrogen-primed OVX rats. LTP could be induced after IA conditioning in the absence of estrogen, but not in its presence (Fig 4C and 4D). However, intact LTP was induced before IA conditioning in estrogen-primed OVX rats (Fig 4A and 4B). Thus, increased estrogen might restrict the capacity for LTP induction after fear conditioning in animals.

### Spine morphology during the estrous cycle

**Fig 5:**
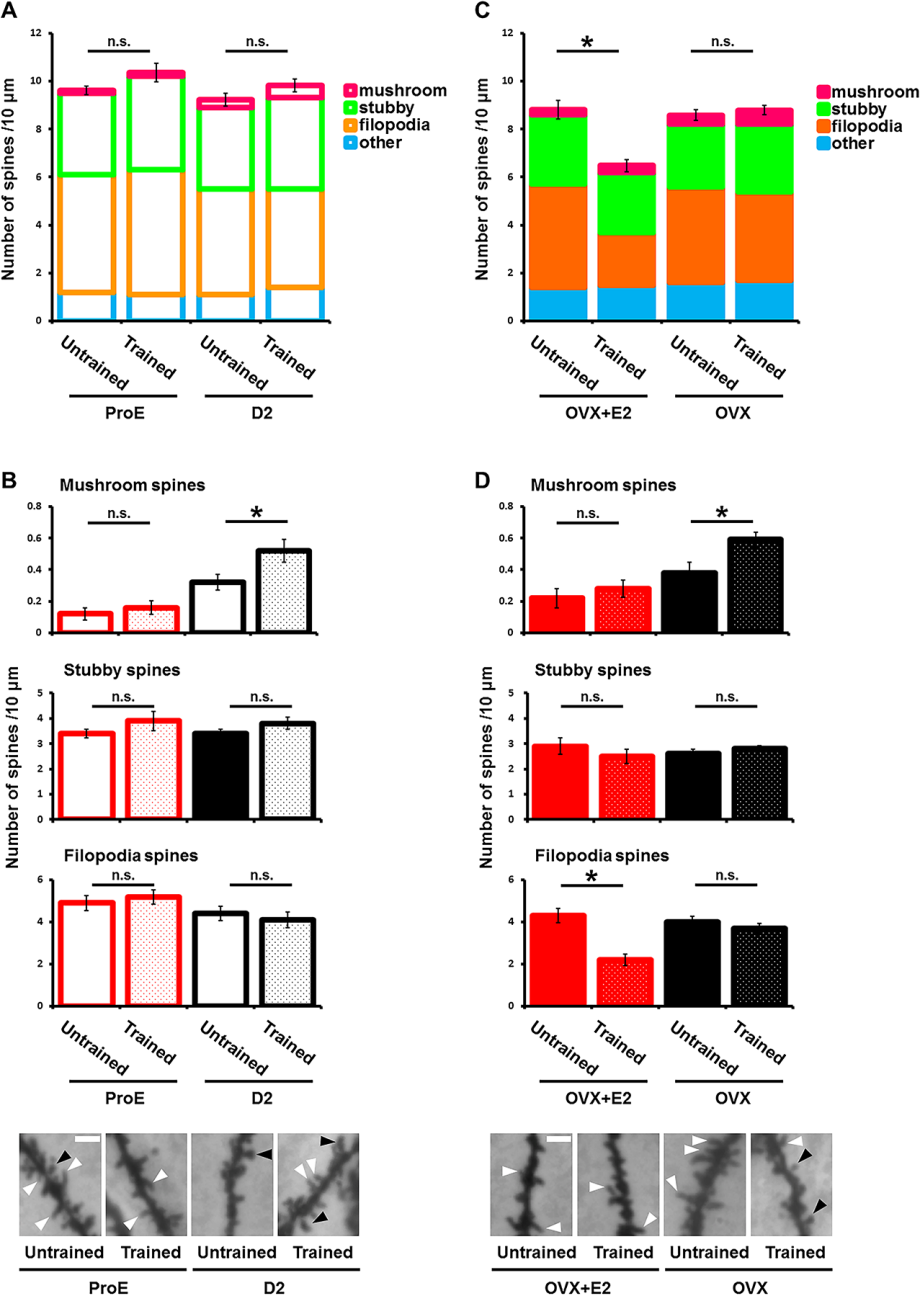
Reduced IA-induced mushroom-spine formation in CA1 pyramidal neurons in ProE rats and E2-primed OVX rats. (A, B) The number of each type of dendritic spine in untrained and IA-trained rats during different periods of the estrous cycle. IA-learning tasks significantly increased the number of mushroom-shaped spines on CA1 pyramidal neurons of D2 rat dendrites (untrained, n = 27; IA-trained, n = 25) but not in ProE rat dendrites (untrained, n = 17; IA-trained, n = 15). (C, D) The number of each type of dendritic spine in untrained and IA-trained rats with or without E2 priming. IA-learning tasks significantly increased the number of mushroom-shaped spines on the dendrites of unprimed OVX rats (untrained, n = 31; IA-trained, n = 44), but not on those of E2-primed OVX rats (untrained, n = 18; IA-trained, n = 20). *P < 0.05, unpaired Student’s *t* test. Error bars represent SEM. (Bottom) Representative photomicrographs (×400) of tertiary apical dendrites of CA1 pyramidal neurons. Black arrowheads indicate mushroom-shaped spines; white arrowheads indicate filopodial spines. Scale bar: 3 μm.

We next investigated spine morphology in the dorsal hippocampal CA1 regions throughout the estrous cycle, in both IA-trained and untrained animals, by Golgi staining. We measured the spine density and the number of mushroom, filopodia, and stubby type spines during the estrous cycle. Spine shape was classified based on a previous study [21]. We found no difference in spine densities between untrained D2 animals and untrained ProE animals (Fig 5A). There was also no difference in spine density between untrained and trained animals, during either the D2 or ProE period (Fig 5A). We found that mature mushroom-type spines were increased by IA learning during D2 (untrained, 0.32 ± 0.05; IA-trained, 0.52 ± 0.07) but not during the ProE period (untrained, 0.12 ± 0.04; IA-trained, 0.16 ± 0.04) (Fig 5B). Consistent with this finding, IA conditioning increased the number of mushroom-type spines in OVX animals without estrogen priming (untrained, 0.38 ± 0.07; IA-trained, 0.59 ± 0.05) but not in estrogen-primed OVX animals (untrained, 0.22 ± 0.06; IA-trained, 0.28 ± 0.06) (Fig 5D). The number of stubby spines and filopodia spines did not differ between IA-trained and untrained intact animals, in either the D2 or ProE periods (Fig 5B). There was no difference in the number of stubby spines before or after IA tasks in OVX animals, with or without estrogen treatment (Fig 5D). The number of filopodia spines was reduced in IA-trained compared to untrained OVX rats when estrogen was present, but not in the absence of estrogen priming (Fig 5D). In contrast to intact animals, the spine density in estrogen-primed OVX animals was decreased by IA training. However, without estrogen priming, IA conditioning did not change the spine density in OVX animals (Fig 5C). This difference in the response of spine density in intact, untreated OVX, and estrogen-treated OVX animals might result from differences in the number of filopodia spines (Fig 5D).

### Additional learning after IA task was prevented during the proestrous period

We next examined whether the attenuation of synaptic plasticity after IA learning during the ProE period prevents the acquisition of additional task learning paradigm. To test this, we conducted a hippocampus-dependent Y-maze task after the IA-learning paradigm. The Y-maze behavioral test is a spatial learning task based on the willingness of rats to explore new environments. We prepared a Y-shaped maze, with three plastic arms set at a 120° angle from each other. The rat was placed at the center of the maze and allowed to freely explore the three arms. The rat exhibits a preference for a previously unexposed arm if the rat remembers the location of the arm. We calculated the rate at which the rat entered an arm other than the most recently visited arm (alternation rate).

**Fig 6:**
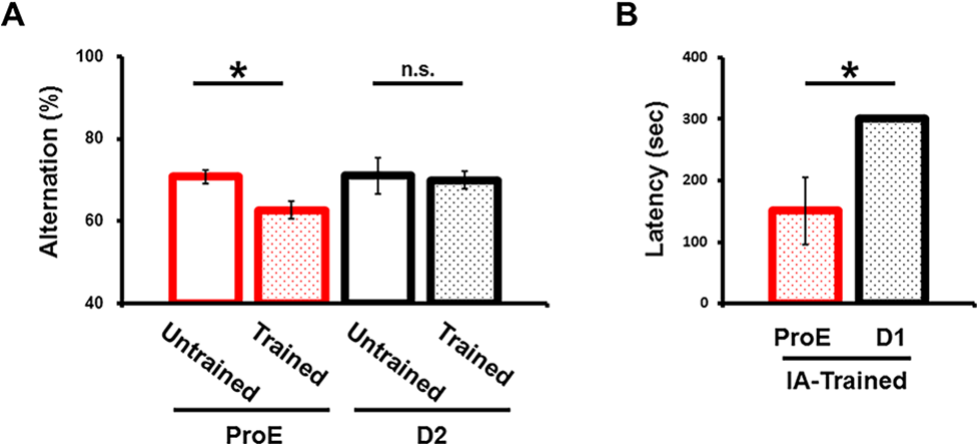
Prior IA-task experience impaired hippocampus-dependent learning in ProE rats. (A) Percentage of spontaneous alternation in the Y-maze task before and after IA training. The alternation rate did not change after IA learning in the D2 period (untrained, n = 8; IA-trained, n = 8), but the alternation rate was reduced after IA learning during the ProE period (untrained, n = 6; IA-trained, n = 10). (B) Latency in the second IA learning session after the initial IA training. The latency to re-enter the dark box in the second IA task was shorter during the ProE period (n = 5) than in the D1 period (n = 4). *P < 0.05, unpaired Student’s *t* test. Error bars represent SEM.

During the ProE period, the alternation rate for Y-maze tests conducted after IA training was lower than that for the tests conducted before IA training (untrained, 70.8% ± 1.6; IA-trained, 62.7% ± 2.2), while there was no difference between the before and after IA training Y-maze alternation rates during other estrous-cycle period (untrained, 71.1% ± 4.5; IA-trained, 70.0% ± 2.2) (Fig 6A). These findings suggest that the experience of IA conditioning attenuates the ability to learn Y-maze task only during the ProE period.

The difference in Y-maze performance after IA conditioning between the ProE period and other period of the estrous cycle was small (Fig 6A). The Y-maze tests working memory, and the contribution of hippocampus to this task could be partial. Thus, we compared the performance of a second IA task, conducted 30 min after the initial IA training, between the ProE period and other periods of the estrous cycle. We found that the latency to re-enter the dark box in the second IA task was shorter during the ProE period than during other period of the estrous cycle (Fig 6B). This finding further suggests that during the ProE period, IA-task experience attenuates successive hippocampus-dependent learning.

## Discussion

Our results suggest that the insertion of CP-AMPARs into the hippocampal CA3-CA1 synapses by the IA task is crucial for the subsequent induction of LTP and the acquisition of additional learning. A transient increase in estrogen prevented the IA-induced synaptic trafficking of CP-AMPARs as well as the induction of LTP after IA conditioning. Thus, the influx of Ca^2+^ through the CP-AMPARs during the initial IA conditioning might be required for the capacity of further plastic changes, including further learning. Previous reports show that an influx of Ca^2+^ through the CP-AMPARs is required for spine enlargement after LTP [22], and that spine enlargement may enhance its capacity to recruit AMPARs [23]. These previous reports are consistent with our results. In this study, we detected an IA-induced increase in CP-AMPARs and in the number of mushroom spines in the dorsal hippocampal CA1 area throughout the estrous cycle, except in the ProE period. Furthermore, IA conditioning increased CP-AMPARs and the number of mushroom type spines in unprimed but not estrogen-primed OVX animals. Thus, the IA-triggered synaptic incorporation of CP-AMPARs could be required for the maturation of spine morphology, leading to LTP induction and the acquisition of additional learning after IA training. We found that this process was inhibited during the ProE period and in estrogen-primed OVX rats. This raises the question of how elevated estrogen levels prevent the IA-induced synaptic incorporation of CP-AMPARs. Previous research shows that the activation of estrogen signaling inactivates cofilin through its phosphorylation by LIM kinase [24]. Since cofilin activation drives AMPAR trafficking [25], the downregulation of cofilin activity by elevated estrogen levels could attenuate the IA-task-induced synaptic delivery of CP-AMPARs.

Although a previous study showed that activation of estrogen receptor improves memory [26], we did not observe any difference in IA performance between unprimed and estrogen-primed OVX animals (Fig 1B). This could be due to our IA-conditioning protocol, which might saturate the effect of the estrogen increase on IA performance.

A previous study reported that the magnitude of LTP is greater in the ProE period than in other periods of the estrous cycle [27], and observed that LTP magnitude measured in the afternoon varied during the estrous cycle, but that the magnitude observed in the morning did not vary. Our LTP experiments were conducted in the morning, and consistent with Warren et al., we did not find any variation in LTP phenotype throughout the estrous cycle in untrained animals. In addition, our LTP-induction protocol differed from that of Warren et al. This could also be the reason why we found no difference in LTP magnitude during the estrous cycle in untrained animals.

A previous study demonstrated that the exogenous application of the estrogen receptor β agonist increased the LTP magnitude [26]. While this study used “untrained animals”, in our study, we induced LTP after IA-training in both untreated and estrogen-primed OVX rats. This difference in experimental conditions may explain the discrepancy in LTP phenotypes in these two studies.

While we found no difference in spine density between IA-conditioned and unconditioned intact animals in the ProE period (Fig 5A), we detected a decrease in spine density (potentially resulting from the difference in the number of filopodia spines between untrained and trained OVX animals primed with estrogen) in IA-conditioned OVX animals primed with estrogen compared to unconditioned ones (Fig 5C). This discrepancy might be due to differences in the pattern of estrogen increase between intact animals and estrogen-primed OVX animals.

Our study delineates the role of the estrous cycle on synaptic plasticity. These findings may shed light on the mental illness such as premenstrual and postmenopausal syndromes.
